# Personalized Privacy-Preserving Frequent Itemset Mining Using Randomized Response

**DOI:** 10.1155/2014/686151

**Published:** 2014-03-30

**Authors:** Chongjing Sun, Yan Fu, Junlin Zhou, Hui Gao

**Affiliations:** Web Science Center, School of Computer Science and Engineering, University of Electronic Science and Technology of China, Chengdu 611731, China

## Abstract

Frequent itemset mining is the important first step of association rule mining, which discovers interesting patterns from the massive data. There are increasing concerns about the privacy problem in the frequent itemset mining. Some works have been proposed to handle this kind of problem. In this paper, we introduce a personalized privacy problem, in which different attributes may need different privacy levels protection. To solve this problem, we give a personalized privacy-preserving method by using the randomized response technique. By providing different privacy levels for different attributes, this method can get a higher accuracy on frequent itemset mining than the traditional method providing the same privacy level. Finally, our experimental results show that our method can have better results on the frequent itemset mining while preserving personalized privacy.

## 1. Introduction

The rapid development of Internet technology, cloud computing, mobile computing, and Internet of things has produced the exploding large datasets, the so-called big data. The data can be used to capture the useful information and interesting patterns and indeed help people to make effective decisions. Data mining is a powerful technique which can discover the hidden interesting patterns and models. By analyzing the data, data mining offers considerable benefits to the consumers, companies, and organizations.

Association rule mining is a popular and important technique of data mining. It is intended to discover the interesting patterns from the large transaction data, that is, frequent patterns and association rules. These rules can be used to improve the market decision making, such as promotional pricing or product placements. For example, by analyzing the transactions data from a supermarket, the rule found that customers who buy diapers also tend to buy beer. Nowadays, association rule mining is employed in many application areas such as Web usage mining, intrusion detection, and bioinformatics.

The process of mining association rules contains two main steps [1]. The first step is to discover all the frequent itemsets with the support being higher than the minimum support. The second step generates the strong rules based on the frequent itemsets found in the first step, and the strong rules have confidence greater than the minimum confidence. In this paper, we focus on the frequent itemset mining, which is the most important part of association rule mining.

With the exponentially increasing information being collected for analysis, there is growing concern about the privacy problem. In fear of the privacy problem, some people might be reluctant to provide the true information. The survey [2] conducted in 1993 demonstrated the worries of people about the privacy. 17% of the respondents are extremely concerned about their personal information. They were reluctant to provide any data and even the privacy protection measures were in place. However, 56% of them were willing to provide their information under the condition that privacy protection methods were adopted. The remaining 27% of them are marginally concerned about their privacy and willing to provide data under any condition. Therefore, in order to collect the true data, privacy protection measures must be taken for protecting the personal information. Besides, before outsourcing or sharing the data with other companies, the data owner also must take some privacy-preserving measures.

Two privacy environments are proposed in [3] based on the privacy mechanisms. The first one is B2B (business to business), which assures that the data obtained from the users would be preprocessed by the privacy-preserving techniques before being supplied to the data miner. The second one is B2C (business to customer), which provides the privacy protection at the point of data collection, that is, at the user site. In order to protect the sensitive information, we proposed methods which can be used under both environments. We focus on the* privacy of input data* in the frequent itemset mining process. Under the B2C environment, the personal data are randomized after each user provides them and then sent to the data collector. Under the B2B environment, the collected dataset is randomized before outsourcing or sharing with others even at public.

Although many works have been proposed to handle the privacy problem in the frequent itemset mining process, few of them focused on the personalized privacy problem. Xiao and Tao [4] proposed the personalized privacy preservation problem considering that different people need different levels of privacy protection, and they used the generalization techniques. Besides, they also raise the problem of the multipublishing [5], wherein different recipients can have different privacy protection levels on the original dataset.

In this paper, we define the personalized privacy as different privacy levels on* different attributes or items* in the process of frequent itemset mining. For example, the customer does not care too much about the disclosure of the information that he bought daily necessities. But he will concern heavily about the privacy problem if he bought some sensitive items. If we provide the same level of privacy protection for different items, the data utility will be lost too much in order to satisfy all items privacy requirements. This is because the nonsensitive items also are disturbed too heavily. Hence, it is necessary to provide the different degree of privacy protection for different items or attributes.

To solve the above personalized privacy problem, we use the randomized response technique [6]. Based on the MASK [7], we give the solution on how to reconstruct the associations under the different privacy protections. Our method can be used to preserve the privacy under both the B2B and B2C environments. Besides, we design a method to accelerate the itemset support computation. Finally, our experimental results show that the performance of our method is better than the traditional method with respect to the accuracy, while satisfying the different privacy requirements.

The rest of this paper is organized as follows. In Section 2, we review the previous related works.  Section 3 gives some related preliminaries and defines the personalized privacy problem. Then in Section 4, we describe the proposed method on the personalized privacy protection and how to discover the frequent itemset from the randomized data. We evaluate our technique in Section 5 by experiments and conclude our work in Section 6.

## 2. Related Works

There are many works on the privacy problem in the association rule mining, and they can be divided into two research streams [3]: input data privacy and output rule privacy. Most of them focus on the second one. To preserve the input data privacy, some methods are adopted to disturb the original data so that the attacker cannot get the true data of users. For the output rule privacy, the collected data are heuristically altered in order to protect some sensitive rules being mined from the dataset by data miners.

The association rule hiding algorithms, which protect the output rule privacy, can be divided into three categories [8], namely, heuristic approaches, border-based approaches, and exact approaches. The first class of approaches selectively sanitizes a set of transactions from the database to hide the sensitive rules efficiently, which suffer from high side effects. Two classic techniques in this class of approaches are distortion [9, 10] and blocking [11, 12]. The second set of approaches [13, 14] hide the sensitive rules by modifying the original borders in the lattice of the frequent and infrequent itemsets in the database. The third set of approaches [15, 16] hide the sensitive rules by solving the Constrain Satisfaction problem using the integer or linear programming. They can find the optimality to hide the rules with high computation cost.

In this paper, we solve the problem of the data input privacy, and the corresponding approaches can be divided into cryptograph-based and reconstruction-based methods. The cryptograph-based approaches handle the problem that some partners want to discover shared association rule from the global data without disclosing their sensitive data to others. The global data may be vertically partitioned [17] or horizontally partitioned [18] and distributed in many partners. The reconstruction-based methods firstly randomize the original data to hide the sensitive data and then reconstruct the interesting patterns based on the statistical features without knowing true values. For the centralized data, there are two distortion strategies,* statistical* distortion and* algebraic* distortion. Rizvi and Haritsa [7] proposed the MASK approach to disturb the original sparse Boolean databases. This method retains each 0 or 1 bit in the database with the probability as *p* and flips this value with the probability as 1 − *p*. Zhang et al. [19] proposed the solution with the algebraic distortion. By using the eigenvalues and eigenvectors, the data of users can be distorted by matrix transformation and noise addition.

Our work focuses on the input data privacy problem of the centralized data. The frequent itemset mining is conducted on the distorted data that is randomized by the randomized response technique. All the above algorithms did not mention the personalized privacy problem that we solve in this paper.

## 3. Preliminaries and Problem Definition

In this section, we introduce the related preliminaries about the privacy-preserving frequent itemset mining and give the problem definition of this paper.

### 3.1. Association Rule Mining

Let *I* be a set of items and *D* the database containing a set of transactions, wherein each transaction *T* is a set of items such that *T*⊆*I*. A transaction *T* is said to contain *X* if and only if *X*⊆*T*. A rule is an implication of the form *X*⇒*Y*, where *X*, *Y* ⊂ *I* and *X*∩*Y* = *ϕ*. The support of the rule *X*⇒*Y* is defined in (1) and its confidence is defined in (2):

(1) $$\begin{array}{c} \mathrm{supp}\left(X⟹Y\right)=P\left(X\cup Y\right), \end{array}$$

(2) $$\begin{array}{c} \text{conf}\left(X⟹Y\right)=P\left(Y\;|\;X\right)=\frac{\mathrm{supp}\left(X\cup Y\right)}{\mathrm{supp}\left(X\right)}, \end{array}$$

where *P*(*X* ∪ *Y*) is the percentage of transactions in *D* that contain *X* ∪ *Y* and supp⁡(*X*) is defined as the percentage of transactions containing *X*.

A set of items *X* is said to be frequent if and only if supp⁡ (*X*) is greater than the user-defined minimum support. Then *X* is a frequent itemset. If the rule *X*⇒*Y* has the support greater than the minimum support and its confidence is greater than the minimum confidence, then *X*⇒*Y* is an interesting rule, that is, association rule. From (1) and (2), it is shown that finding the association rules is effectively equivalent to generating all the frequent itemsets with support greater than the minimum support. Therefore, we focus on the frequent itemset mining.

### 3.2. Randomized Response

Randomized response technique was first introduced by Warner [20] to solve the statistical survey problem of the sensitive questions. For example, social scientists want to know how many people in some area use drugs. Usually, respondents are reluctant to directly answer this kind of questions. Hence, Warner proposed the randomized response.

To ask a binary choice question about whether people have a sensitive attribute *A*, the randomized response gives two related questions like the following:

1. I have the sensitive attribute *A*;
2. I do not have the sensitive attribute *A*.

Respondents decide which question to answer with a probability *p*. Then the interviewer only gets a “yes” or “no” without knowing which question the respondent answered. If a respondent has the sensitive attribute *A*, then he will give the answer “yes” to answer the first question with probability as *p* or “no” with probability as 1 − *p*. If a person does not have *A*, then he will give “no” with probability as *p* to answer the first question and “yes” with probability as 1 − *p* to answer the second question. Hence the probability that an interviewer gets the answer “yes” can be computed by (3) while getting “no” can be computed by (4):

(3) $$\begin{array}{c} P\left(\text{ans}=\text{yes}\right)=P\left(A=\text{yes}\right)\cdot p+P\left(A=\text{no}\right)\cdot \left(1-p\right), \end{array}$$

(4) $$\begin{array}{c} P\left(\text{ans}=\text{no}\right)=P\left(A=\text{no}\right)\cdot p+P\left(A=\text{yes}\right)\cdot \left(1-p\right), \end{array}$$

where *P* (*A* = yes) is the proportion of the respondents that have the attribute *A*, while *P* (*A* = no) is the proportion of respondents that do not have the attribute *A*. The interviewer can get the proportion of respondents having the attribute *A*, *P* (*A* = yes), by solving (3) and (4).

### 3.3. Problem Definition

In this paper, we solve the data privacy problem with respect to the frequent itemset mining under the B2B environments as well as the B2C environment.

Under the B2C model, the interviewer can conduct a survey containing sensitive questions. For example, the questions on “whether you are divorced,” “whether you have criminal records,” or “whether you use drugs” are sensitive. This kind of surveys can analyze the association between factors and indeed reflect sociology problems. In this model, respondents disturb survey vectors with the given parameters and then send them to the reviewers.

Under the B2B model, the data owner such as supermarket managers disturbs the original data before sharing with other business partners. Taking the market transactions as an example, customers are reluctant to let others know what he or she bought, especially some sensitive products. By taking the privacy protection measures, transaction details can be hidden without leaking the personal privacy.

Considering the data utility and different degree of privacy requirements, the protection on the different attributes or items should be different. For example, the customer does not care too much about the disclosure that he bought papers, while concerning heavily on some sensitive items.

The problem of* personalized privacy-preserving frequent itemset mining* is, given the original transaction dataset *D*, how to disturb *D* into *D*′ satisfying the different privacy requirements and mine frequent itemsets from *D*′, so that the frequent itemsets mined from *D* are close to the frequent itemsets mined from *D*′ as much as possible.

## 4. Personalized Privacy-Preserving Frequent Itemset Mining

In this section, we present the procedure on how to distort the original data by the randomized response technique satisfying the personalized privacy requirement. Then, we give the method to reconstruct itemset support from the distorted data. Finally, we devise a personalized privacy-preserving frequent itemset mining algorithm based on the Apriori algorithm.

### 4.1. Personalized Data Distortion

Suppose there are *n* items in the database *D*. Each transaction in *D* is represented by a binary vector *T*. *T*_*i*_ = 1 if this transaction contains the item *i*; otherwise, *T*_*i*_ = 0. For each item *i*, 1 ≤ *i* ≤ *n*, there is a probability parameter *p*_*i*_  (0 ≤ *p*_*i*_ ≤ 1) to disturb the data on the item *i*, and these parameters form a vector shown in formula (5). Then the distorted value of this transaction on item *i* can be expressed in (6), where *r*_*i*_ is a value randomly drawn from a uniform distribution over the interval [0,1]:

(5) $$\begin{array}{c} P=\left(p_{1},p_{2},\ldots ,p_{n}\right), \end{array}$$

(6) $$\begin{array}{c} T_{i}^{\prime }=\{\begin{array}{cc} T_{i}, & r_{i}\leq p_{i},\\ 1-T_{i}, & \text{otherwise}. \end{array} \end{array}$$

From (6) we can see that the *i*th value of transaction *T* is kept with probability *p*_*i*_ and flipped with probability 1 − *p*_*i*_. The proportion of *T*_*i*_′ = 1 can be calculated by (7), and the proportion of *T*_*i*_′ = 0 can be calculated by (8):

(7) $$\begin{array}{c} P\left(T_{i}^{\prime }=1\right)=P\left(T_{i}=1\right)\cdot p_{i}+P\left(T_{i}=0\right)\cdot \left(1-p_{i}\right), \end{array}$$

(8) $$\begin{array}{c} P\left(T_{i}^{\prime }=0\right)=P\left(T_{i}=0\right)\cdot p_{i}+P\left(T_{i}=1\right)\cdot \left(1-p_{i}\right). \end{array}$$

For the personalized privacy requirements, different items are distorted with different probability parameters. For a given parameter *p*_*i*_, the probability of correct reconstruction on the value *T*_*i*_ = 1 is calculated by

(9) R(pi,si)=P(Ti′=1 ∣ Ti=1)·P(Ti=1 ∣ Ti′=1)+P(Ti′=0 ∣ Ti=1)·P(Ti=1 ∣ Ti′=0).

The first part in (9) means that the original value *T*_*i*_ = 1 is distorted into value *T*_*i*_′ = 1 and then reconstructed from *T*_*i*_′ = 1, and the second part is for the distorted value *T*_*i*_′ = 0. Finally, the probability can be computed by (10), where *s*_*i*_ is the support of item *i* in original database. The similar derivation of (10) can be found in [7]:

(10) $$\begin{array}{c} R\left(p_{i},s_{i}\right)=\frac{s_{i}p_{i}^{2}}{s_{i}p_{i}+\left(1-s_{i}\right)\left(1-p_{i}\right)}+\frac{s_{i}{\left(1-p_{i}\right)}^{2}}{s_{i}\left(1-p_{i}\right)+\left(1-s_{i}\right)p_{i}}. \end{array}$$

**Figure 1:**
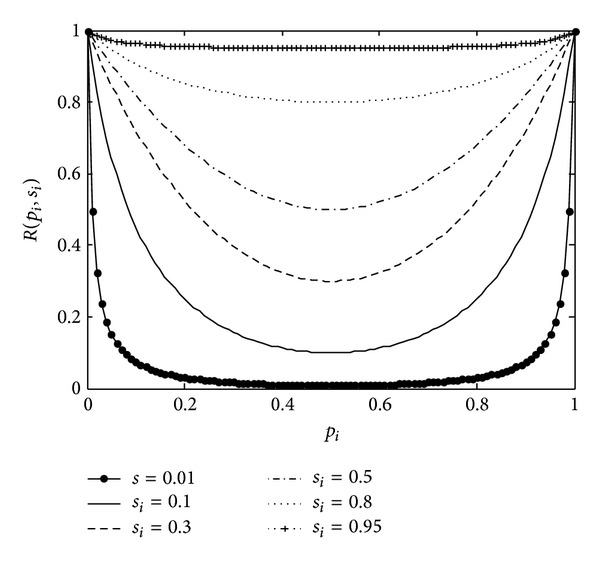
Reconstruction probability.

The reconstruction probability curves of (10) are shown in Figure 1. We can see that the higher the item support is, the easier this item having value 1 will be reconstructed. Besides, the curves are symmetric around *p*_*i*_ = 0.5. The further the distance between *p*_*i*_ and 0.5, the easier the item having value 1 will be reconstructed. Therefore, for a given item, the different probability parameters will lead to the different degree of privacy protection. The parameters for items will be set by the data owner according to the properties of these items. For example, the parameter *p*_*i*_ for the item milk can be very high even 1.0, while for the drugs it can be more close to 0.5.

Under the B2C environment, the interviewer and the respondents cooperate with each other to set the parameter vector *P*. Then respondents disturb their personal data and send them to the interviewer. While under the B2B environment, the data owner disturbs the original data with parameter vector *P* and sends the distorted data with *p* to the third parties.

### 4.2. Itemset Support Reconstruction

After getting the distorted data *D*′, the data mining will reconstruct the support for itemsets to find the frequent ones. In order to reconstruct the support of an itemset, we need to get the count of every combination of the items in this itemset. For example, in order to compute the support of itemset *ABC* in original dataset *D*, that is, *p* (*A* = 1, *B* = 1, *C* = 1), we compute the count of 2^3^ combinations of *ABC* in *D*′, that is, *c*′(*A* = 0, *B* = 0, *C* = 0), *c*′(*A* = 0, *B* = 0, *C* = 1),…, and *c*′(*A* = 1, *B* = 1, *C* = 1). This is because the original value (*A* = 1, *B* = 1, *C* = 1) can be distorted to these 2^3^ combinations of *ABC*.

Let *S* be the combinations of the *k* items in (11), where *S*_*j*_ is the binary form of value *j* in (12). For example, when *k* = 3 with items *ABC*, *S*_0_ is (*A* = 0, *B* = 0, *C* = 0); *S*_3_ is (*A* = 0, *B* = 1, *C* = 1); that is, 011 is the binary form of 3:

(11) $$\begin{array}{c} S=\left\{S_{0},S_{1},\ldots ,S_{2^{k}-1}\right\}, \end{array}$$

(12) $$\begin{array}{c} S_{i}=\text{binary}\left(i,k\right). \end{array}$$

The combination *S*_*i*_ in the original database can be distorted into any combination *S*_*j*_ in *S*. According to the probability theory, the probability of *S*_*i*_ being distorted to *S*_*j*_ can be computed by

(13) $$\begin{array}{c} r_{ij}=\text{prod}\left(\left(S_{i}⊙S_{j}\right)\circ P^{\left(k\right)}+\left(S_{i}⊕S_{j}\right)\circ \left(1-P^{\left(k\right)}\right)\right), \end{array}$$

where ∘ is the Hadamard product of two vectors and *P* ^(*k*)^ is the distorting probability parameters corresponding to the *k* items. prod() returns the product of the vector elements. That means that if the value on an item *i* is kept, then the *p*_(*i*)_ is multiplied to *r*_*ij*_, otherwise 1 − *p*_(*i*)_. An example with *k* as 3 in Table 1 illustrates the formulation on the transition matrix.

Corresponding to the combinations defined in (11) of the given *k* items, we define their counts in the original database *D* and the distorted database *D*′, respectively, in (14), where *C* is the count vector for *D*, while *C*′ is the count vector for *D*′. *c*_*i*_ and *c*_*i*_′ are the counts of the combination *S*_*i*_ for the *k* items in *D* and *D*′, respectively:

(14) $$\begin{array}{c} C=\left[\begin{array}{c} c_{0}\\ c_{1}\\ \vdots \\ c_{2^{k}-1} \end{array}\right],\;\;C^{\prime }=\left[\begin{array}{c} c_{0}^{\prime }\\ c_{1}^{\prime }\\ \vdots \\ c_{2^{k}-1}^{\prime } \end{array}\right]. \end{array}$$

Then the relationship between *C* and *C*′ can be expressed by (15) according to the randomized response distortion mechanism:

(15) $$\begin{array}{c} C^{\prime }=RC. \end{array}$$

After getting the transition matrix *R* with elements computed by (12), we can reconstruct the counts of combinations by (16). Hence, we evaluate the performance of the reconstruction by computing the difference between *C* and $\hat{C}$:

(16) $$\begin{array}{c} \hat{C}=R^{-1}C^{\prime }. \end{array}$$

The count of the last combination divided by the number of transactions is* the estimated support* of the corresponding itemset as shown in (17), where *m* is the number of transactions in *D*:

(17) $$\begin{array}{c} \hat{s}=\frac{{\hat{c}}_{2^{k}-1}}{m}. \end{array}$$

### 4.3. Mining Frequent Itemset from Distorted Dataset

We mine the frequent itemset from the database by using the classic Apriori algorithm, which employs an iterative approach to search the frequent itemset. The frequent *k*-itemsets are used to generate the candidate (*k* + 1)-itemsets by the* AprioriGen*. Then Apriori computes the support of candidate (*k* + 1)-itemsets and filters out the itemsets with support less than the minimum support. These operations iteratively run until no more frequent itemsets can be found.

In the privacy-preserving personalized frequent itemset mining, we need to reconstruct the support of candidate itemsets from the distorted database. For a candidate itemset, we compute the counts of all its items combinations. In order to accelerate the counting speed, we firstly remove the items with support less than the minimum support. Then for the candidate itemset *X* = {*I*_1_,…, *I*_*k*_}, we extract the subdataset *D*(*X*) = *D*(*I*_1_,…, *I*_*k*_) from the database *D*′. In the subdataset *D*(*X*), we map each transaction *t* ∈ *D*(*X*) into a value by

(18) $$\begin{array}{c} v=t\cdot b=\left(t_{1},t_{2},\ldots ,t_{k}\right)\cdot \left(2^{k-1},2^{k-2},\ldots ,2^{0}\right). \end{array}$$

**Figure 2:**
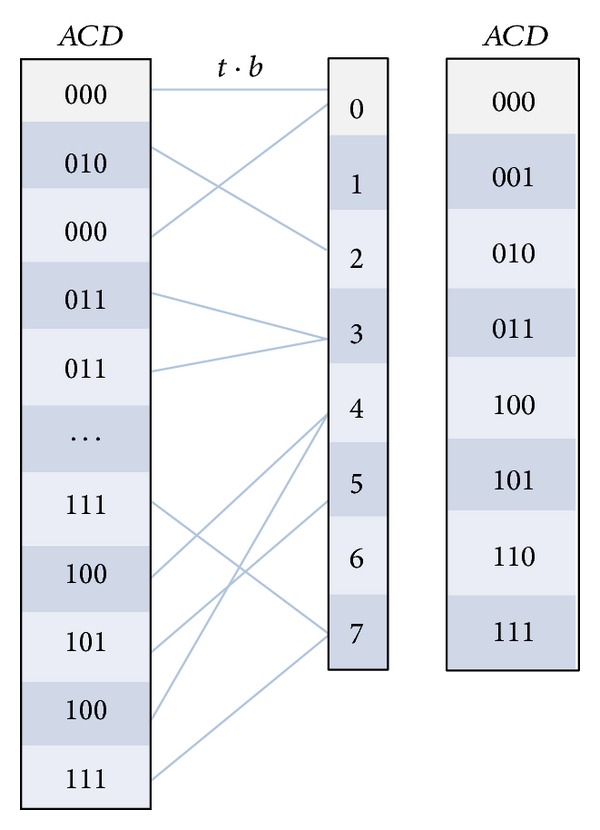
An example of transaction mapping.

By (18), a vector *V* will be computed, wherein *v*_*i*_ = *D*(*X*)*i* · *b*. Then *c*_*j*_′ in (14) is the count of elements in *V* equal to *j*. By this method, it is very fast to compute the vector *C*′.  Figure 2 gives an example of transaction mapping that maps the transaction *t* into a value. By computing the count of each value from 0 to 7, we can get the counts of all the combinations for the itemset *ACD*. By using (16) and (17), we can get the support of the itemset *X*.

In our privacy-preserving personalized frequent itemset mining, we iteratively generate the candidate (*k* + 1)-itemset from the frequent *k*-itemset and check the support of the candidate itemset using the itemset support reconstruction.

## 5. Experimental Results

In this section, we evaluate the performance about the personalized privacy-preserving frequent itemset mining on the real dataset and synthetic datasets generated by the classic IBM Quest Market-Basket Synthetic Data Generator. (The C++ source code can be downloaded at http://www.cs.loyola.edu/~cgiannel/assoc_gen.html).

### 5.1. Datasets

The synthetic datasets are generated by the IBM Almaden generator with the parameters TIDN [1], where *T* is the average size of the transactions, *I* is the average size of the maximal potentially large itemsets, *D* is the number of transactions, and *N* is the number of items. We generated two synthetic datasets T3.I4.D500K.N10 and T40.I10.D100K.N942.

The real dataset is BMS-WebView-1 [21], which contains the click-stream data from the website of a legwear and leg care retailer. The dataset contains 59,602 transactions and 497 items. We scaled the dataset with a factor of 10 and got the dataset BMS-WebView-1x10, which contains 596,020 transactions and 497 items.

### 5.2. Evaluation Metrics

We evaluate our method on the distorted database by two kinds of error metrics [7], the support errors and the identity error. Let *F* be the frequent itemsets discovered from the original database *D* by the Apriori algorithm and $\hat{F}$ represent the reconstructed frequent itemset minded from the distorted database by our method.

The* support error* metric is evaluated by (19), which reflects the relative error in the support values. We measure the average error based on the frequent itemsets which are correctly identified to be frequent, that is, $F\cap \hat{F}$, with the given minimum support *s*_min⁡_:

(19) $$\begin{array}{c} \rho =\frac{1}{\left|F\cap \hat{F}\right|}\sum_{f\in F\cap \hat{F}}\frac{\left|{\hat{s}}_{f}-s_{f}\right|}{s_{f}}. \end{array}$$

The* identity error* metrics are given in (20). *σ* ^+^ indicates the percentage of false positive, that is, the percentage of reconstructed itemsets which do not exist in the original frequent itemsets. *σ* ^−^ indicates the percentage of false negative, that is, the percentage of original frequent itemsets which are not correctly reconstructed as frequent:

(20) $$\begin{array}{c} \sigma^{+}=\frac{\left|\hat{F}\right|-\left|F\cap \hat{F}\right|}{\left|F\right|},\\ \sigma^{-}=\frac{\left|F\right|-\left|F\cap \hat{F}\right|}{\left|F\right|}. \end{array}$$

**Figure 3:**
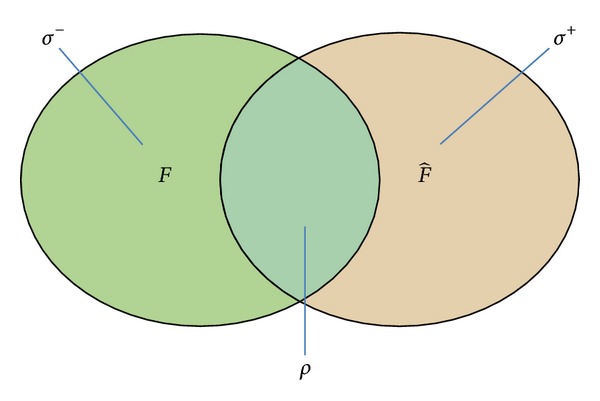
Evaluation metrics.

The corresponding metrics are illustrated in Figure 3. For the part of metric *ρ*, the intersection of two frequent itemsets *F* and $\hat{F}$ is maybe empty. Under this condition, we do not compute the result of *ρ*.

### 5.3. Results Analysis

We evaluate our method by measuring the support error and the two identity errors shown in formulas (19), (20), and we conduct our experiments on the three given datasets described in Section 5.1. We compare our method with* MASK* which distorts the original dataset with only one parameter value *p*. If the personalized distorted vector of our method is *P*, then *p* = min⁡(*P*). In our experiments, the elements in the personalized distorted vector *P* in formula (5) are generated following the uniform distribution with the range of [0.8, 0.95]. The average value of vector *P* is 0.8741. In order to protect all the items or attributes in a dataset, the parameter *p* in MASK must be set as 0.8. Therefore, we compare our method with the MASK having the parameter *p* as 0.8.

#### 5.3.1. Comparisons with Different Minimum Support

**Figure 4:**
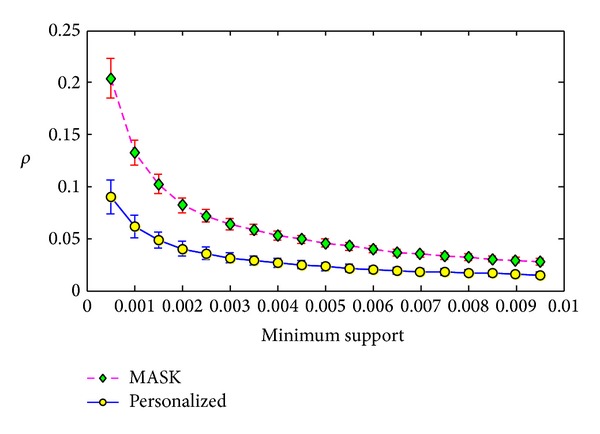
Support error *ρ* versus minimum support on T3.I4.D500K.N10.

**Figure 5:**
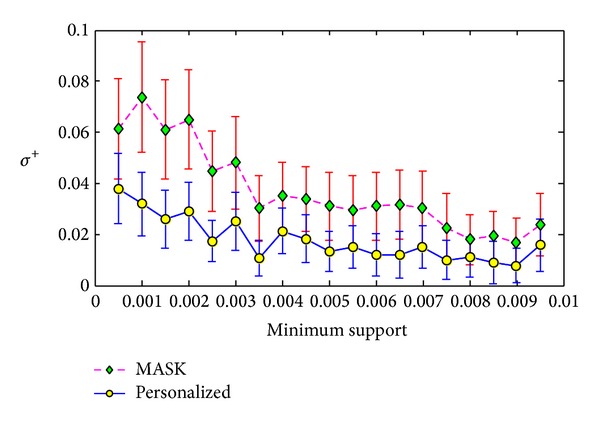
Frequent itemset added ration *σ* ^+^ versus minimum support on T3.I4.D500K.N10.

**Figure 6:**
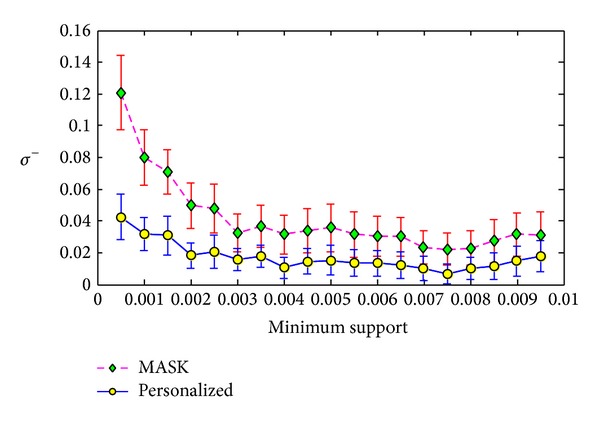
Frequent itemset lost ration *σ* ^−^ versus minimum support on T3.I4.D500K.N10.

The first experiment was conducted on the T3.I4.D500K.N10. In this experiment, we set the minimum support from 0.05% to 0.95% with the step length as 0.05%. For each minimum support, we ran the MASK and our personalized method 100 times and compute the average values and the standard deviations of evaluation metrics. The results are shown in Figures 4, 5, and 6.

From Figures 4, 5, and 6, we can see that our method can have smaller error than MASK. This is because the MASK has to distort all the items or attributes in the maximum distorting level in order to protect each item or attribute, while our method distort different items with different distorting level. The results show that our method only leads to half of support error *ρ* of MASK in the dataset T3.I4.D500K.N10. Besides, from Figures 5 and 6, we can see that our method can have much smaller deviations of identity errors.

We conducted the same experiments on the real dataset BMS-WebView-1x10. For this dataset, we set the minimum support from 0.2% to 0.55% with the step length as 0.05%, and for each minimum support, we ran the algorithms 20 times. As the average value of elements in vector *P* is 0.8741, we also ran the MASK with parameter *p* as 0.8741. Besides, we ran the MASK with *p* as the maximum value of *P*, that is, 0.95.

**Figure 7:**
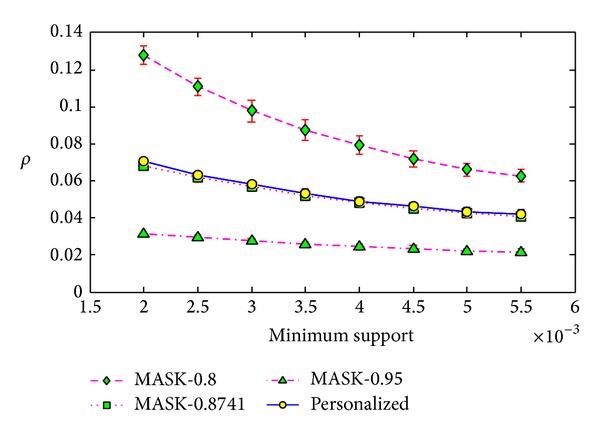
Support error *ρ* versus minimum support on BMS-WebView-1x10.

**Figure 8:**
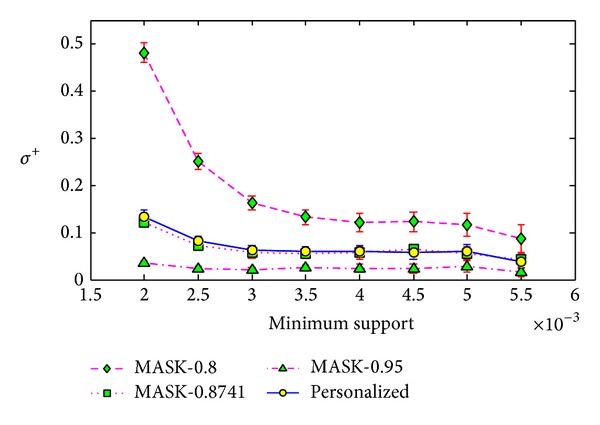
Frequent itemset added ration *σ* ^+^ versus minimum support on BMS-WebView-1x10.

**Figure 9:**
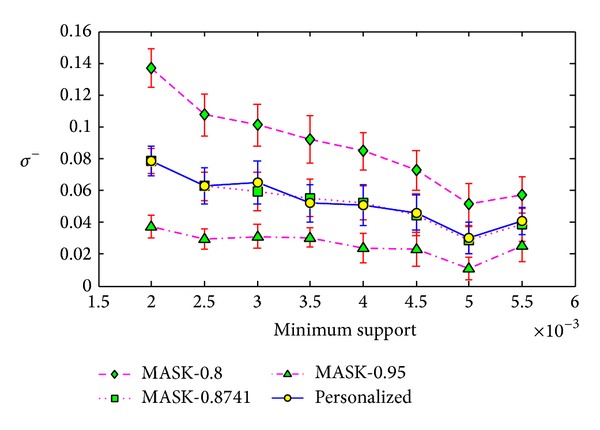
Frequent itemset lost ration *σ* ^−^ versus minimum support on BMS-WebView-1x10.

We show the results in Figures 7, 8, and 9, and the results here show that our method is much better than MASK with *p* as 0.8. However, the results of our method are much similar to the results of MASK with *p* as 0.8741, the average value of vector *P* for our method. Similar results can be found in Figures 13, 14, and 15 on the dataset T40.I10.D100K.N942. But the MASK with *p* as 0.8741 cannot protect all the items or attributes, because some attribute needs protection with privacy level smaller than 0.8741, such as 0.85. For the three metrics, the results of MASK with *p* as 0.95 are better than our method. This is because the privacy protection level of each attribute in our method is between 0.8 and 0.95.

#### 5.3.2. The Impact of the Size of Dataset

We conduct the second experiment to evaluate the impact of the size of dataset on the reconstruction errors shown in formulas (19), (20). The dataset BMS-WebView-1x10 is formed from the dataset BMS-WebView-1 by copying it 10 times. Similarly, we set the minimum support of frequent itemset mining from 0.2% to 0.55% with the step length as 0.05% and run the algorithms 20 times for each minimum support.

**Figure 10:**
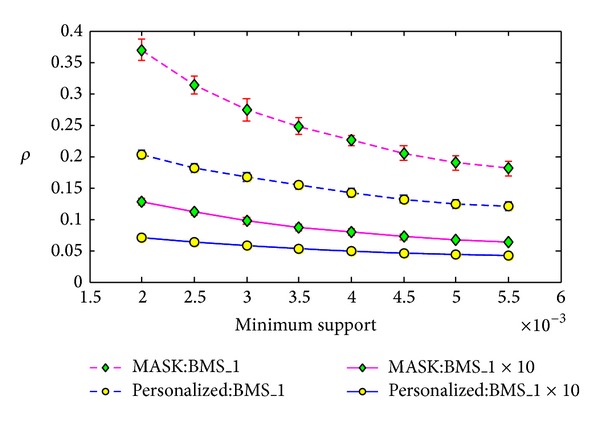
Support error *ρ* versus minimum support on different size of datasets.

**Figure 11:**
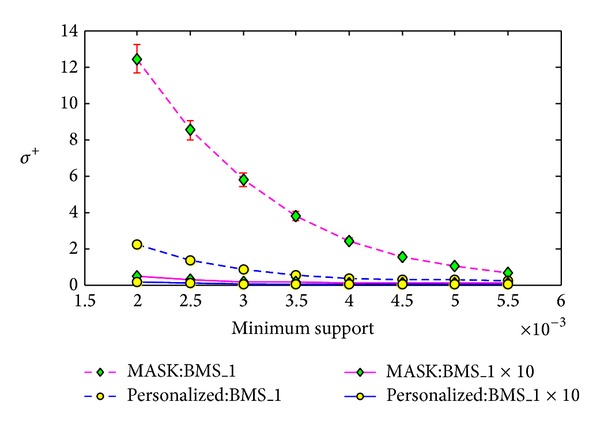
Frequent itemset added ration *σ* ^+^ versus minimum support on different size of datasets.

**Figure 12:**
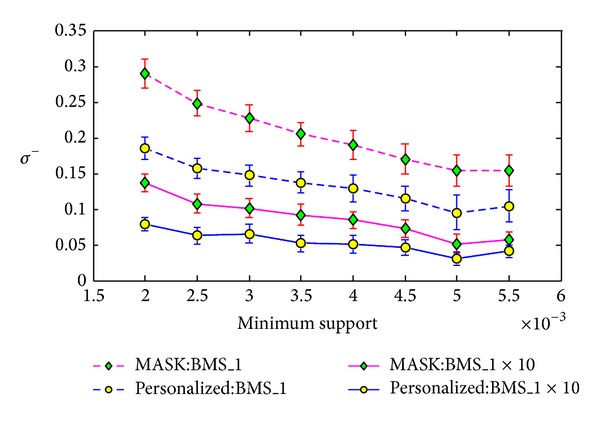
Frequent itemset lost ration *σ* ^−^ versus minimum support on different size of datasets.

The corresponding results in Figures 10, 11, and 12 show that the reconstruction errors on BMS-WebView-1x10 are much smaller than the errors on BMS-WebView-1 for both MASK and our method. Note that the frequent itemsets discovered from BMS-WebView-1 are the same as the frequent itemsets discovered from BMS-WebView-1x10. This gives us a thought that if we want to improve the reconstruction results, we can copy the original dataset many times and distort the new dataset and send it to the cooperated party. Then the cooperated party can accurately discover the interesting patterns without knowing the original private information.

#### 5.3.3. Comparisons with Different Lengths of Frequent Itemset

The third experiment is conducted on the dataset T40.I10.D100K.N942. In this experiment, we set the minimum support as 1.45%, and we run the corresponding algorithms 5 times and get the average values. We also set the parameter *p* of MASK as 0.8, 0.8741, and 0.95 separately.

**Figure 13:**
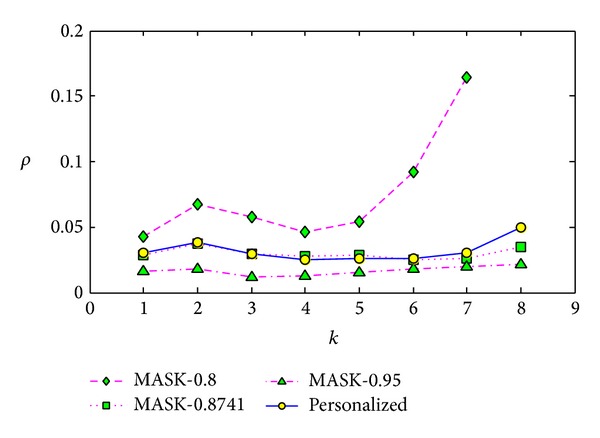
Support error *ρ* versus itemset length *k* on dataset T40.I10.D100K.N942.

**Figure 14:**
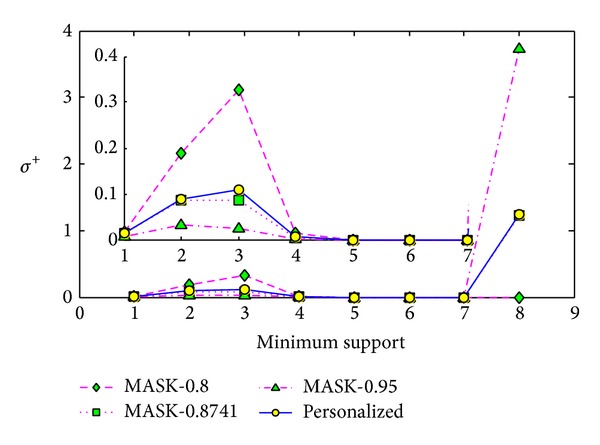
Frequent itemset added ration *σ* ^+^ versus itemset length *k* on dataset T40.I10.D100K.N942.

**Figure 15:**
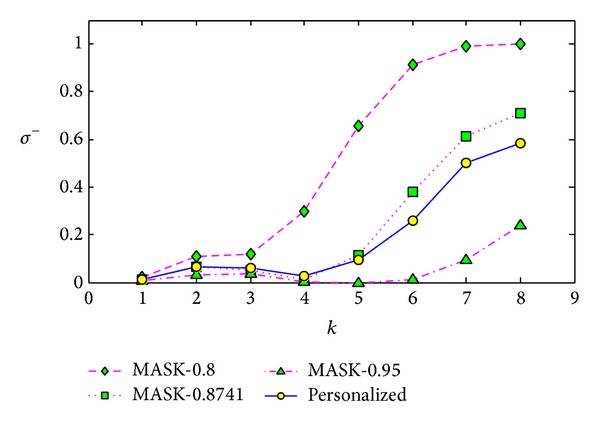
Frequent itemset lost ration *σ* ^−^ versus itemset length *k* on dataset T40.I10.D100K.N942.

For the minimum support 1.45%, the maximum length of original frequent itemsets is 8, and the number of frequent *k*-itemset is shown in Table 2. Then we compare the result on each itemset length from 1 to 8. The results shown in Figures 13, 14, and 15 demonstrate that our method performs better than MASK with *p* as 0.8 for each itemset length and performs very similar to the MASK with *p* as 0.8741.

In Figure 13, as the intersection set of frequent 8-itemsets reconstructed by MASK with *p* as 0.8 and original frequent 8-itemsets is an empty set, the support error cannot be computed; then the value cannot be shown. We cannot conclude the clear trend of the reconstruction error with the itemset length increasing. In Figure 14, when *k* is 8, the frequent itemset added ratio increased sharply. By carefully analyzing the results, we found that many new frequent itemsets have the support very close to the minimum support, which leads to the heavy identity error.

## 6. Conclusions

In this paper, we solve the problem of how to provide the personalized privacy protection on different attributes or items while discovering the frequent itemsets. Based on the classic randomized response technique, we proposed a personalized privacy-preserving method. Besides, we proposed a method to improve the efficiency of counting the frequent itemsets by mapping the frequent itemset vector into a value. Experimental results show that the personalized privacy protection method can have much better performance than the traditional privacy protection method which provides the same privacy protection for the different items. From the experimental results, we can see that we can copy the original dataset many times to create a new dataset and then distort this new dataset. Then the others can discover the frequent itemsets with smaller error at the expense of more computation and communication cost.

## Figures and Tables

**Table 1:** An example of transition matrix.

	000	⋯	110	111
000	*p*_1_ *p*_2_ *p*_3_	⋯	(1 − *p*_1_)(1 − *p*_2_)*p*_3_	(1 − *p*_1_)(1 − *p*_2_)(1 − *p*_3_)
001	*p*_1_ *p*_2_(1 − *p*_3_)	⋯	(1 − *p*_1_)(1 − *p*_2_)(1 − *p*_3_)	(1 − *p*_1_)(1 − *p*_2_)*p*_3_
${\begin{array}{c} \vdots \end{array}}^{}$	⋮	⋮	⋮	⋮
111	(1 − *p*_1_)(1 − *p*_2_)*p*_3_	⋯	*p*_1_ *p*_2_(1 − *p*_3_)	*p*_1_ *p*_2_ *p*_3_

**Table 2:** The number of frequent *k*-itemsets.

*k*	1	2	3	4	5	6	7	8
|*F*|	690	4869	293	368	483	469	331	25

## References

[1] Agrawal R, Srikant R Fast algorithms for mining association rules.

[2] Cranor LF, Reagle J, Ackerman MS (2000). *Beyond Concern: Understanding Net Users' Attitudes about Online Privacy*.

[3] Haritsa JR (2008). Mining association rules under privacy constraints. *Privacy-Preserving Data Mining*.

[4] Xiao X, Tao Y Personalized privacy preservation.

[5] Xiao X, Tao Y, Chen M (2009). Optimal random perturbation at multiple privacy levels. *Proceedings of the VLDB Endowment*.

[6] Tamhane AC (1981). Randomized response techniques for multiple sensitive attributes. *Journal of the American Statistical Association*.

[7] Rizvi SJ, Haritsa JR Maintaining data privacy in association rule mining.

[8] Verykios VS, Gkoulalas-Divanis A (2008). A survey of association rule hiding methods for privacy. *Privacy-Preserving Data Mining*.

[9] Wu Y-H, Chiang C-M, Chen ALP (2007). Hiding sensitive association rules with limited side effects. *IEEE Transactions on Knowledge and Data Engineering*.

[10] Pontikakis ED, Tsitsonis AA, Verykios VS (2004). An experimental study of distortion-based techniques for association rule hiding. *Research Directions in Data and Applications Security XVIII*.

[11] Pontikakis ED, Theodoridis Y, Tsitsonis AA, Chang L, Verykios VS A quantitative and qualitative analysis of blocking in association rule hiding.

[12] Wang S-L, Jafari A Using unknowns for hiding sensitive predictive association rules.

[13] Xingzhi S, Yu PS A border-based approach for hiding sensitive frequent itemsets.

[14] Moustakides GV, Verykios VS A max-min approach for hiding frequent itemsets.

[15] Menon S, Sarkar S, Mukherjee S (2005). Maximizing accuracy of shared databases when concealing sensitive patterns. *Information Systems Research*.

[16] Gkoulalas-Divanis A, Verykios VS An integer programming approach for frequent itemset hiding.

[17] Vaidya J, Clifton C Privacy preserving association rule mining in vertically partitioned data.

[18] Kantarcioglu M, Clifton C (2004). Privacy-preserving distributed mining of association rules on horizontally partitioned data. *IEEE Transactions on Knowledge and Data Engineering*.

[19] Zhang N, Wang S, Zhao W (2004). A new scheme on privacy preserving association rule mining. *Knowledge Discovery in Databases: PKDD 2004*.

[20] Warner SL (1965). Randomized response: a survey technique for eliminating evasive answer bias. *Journal of the American Statistical Association*.

[21] Zheng Z, Kohavi R, Mason L Real world performance of association rule algorithms.

